# Development and validation of an interprofessional protocol for high-risk prenatal care

**DOI:** 10.1590/0034-7167-2023-0197

**Published:** 2025-03-10

**Authors:** Daniella Carvalho Araújo, Láisa Rebecca Sousa Carvalho, Leyla Gerlane de Oliveira Adriano, Ravena de Sousa Alencar Ferreira, Girzia Sammya Tajra Rocha, Priscila de Souza Aquino, Victórya Suéllen Maciel Abreu, Herla Maria Furtado Jorge

**Affiliations:** I Universidade Federal do Piauí. Teresina, Piauí, Brazil; II Universidade Federal do Ceará. Fortaleza, Ceará, Brazil

**Keywords:** Validation Study, Health Research Evaluation, Prenatal Care, Pregnancy High-Risk, Interdisciplinary Health Team, Estudio de Validación, Atención Prenatal, Embarazo de Alto Riesgo, Protocolo, Equipo Interdisciplinario de Salud

## Abstract

**Objectives:**

to develop and validate the content of an interprofessional care protocol for high-risk prenatal care.

**Methods:**

this methodological study employed a quantitative approach and was conducted from June 2022 to February 2023. The first phase involved developing the protocol using the Convergent Care Research methodology, while the second phase consisted of content validation by 11 expert judges. The Content Validity Index (CVI) ≥ 0.80 was applied to assess the degree of agreement among the judges, and Cronbach’s alpha was used to evaluate the instrument’s reliability.

**Results:**

content validation was achieved with an overall CVI of 0.990, and internal validation showed a Cronbach’s alpha of 0.889.

**Conclusions:**

the protocol is validated in terms of content, with a high level of recommendation from the judges, which will enhance the quality of high-risk prenatal care.

## RESUMEN

**Objetivos:**

construir y validar el contenido de un protocolo asistencial interprofesional para el cuidado prenatal en embarazos de alto riesgo.

**Métodos:**

estudio metodológico con enfoque cuantitativo, realizado de junio de 2022 a febrero de 2023. La primera fase del estudio consistió en la elaboración del protocolo mediante la Investigación Convergente Asistencial, y en la segunda, se llevó a cabo la validación de contenido a través de la evaluación del protocolo por 11 jueces especialistas. Se utilizó el Índice de Validez de Contenido (IVC) ≥ 0,80 para el análisis del grado de concordancia entre los jueces, y el alfa de Cronbach para la confiabilidad del instrumento.

**Resultados:**

se alcanzó la validación de contenido del protocolo con un IVC general de 0,990 y una validación interna con un alfa de Cronbach de 0,889.

**Conclusiones:**

el protocolo es válido en cuanto a su contenido, con un alto grado de recomendación por parte de los jueces, lo que promoverá mejoras en la calidad de la atención prenatal en embarazos de alto riesgo.

## INTRODUCTION

Attention to high-risk prenatal care involves specific characteristics and needs, particularly regarding guidance on the safest type of delivery in each situation^(1)^. Approximately 830 women die every day from pregnancy- or childbirth-related complications worldwide. In 2017, there were around 295,000 maternal deaths during and after pregnancy and childbirth, mostly in low-resource settings, with cases considered preventable. Maternal health care gained global prominence as one of the Millennium Development Goals established by the World Health Organization in 2000. This milestone reshaped global priorities, with the goal of reducing the global maternal mortality rate to fewer than 70 deaths per 100,000 live births^(2)^.

In Brazil, public policies have been implemented since the beginning of this millennium, such as the development of the Humanization Program for Prenatal and Childbirth Care, aimed at improving obstetric quality and reducing maternal and perinatal mortality^(3,4)^. The Ministry of Health’s Ordinance GM/MS No. 13, issued in January 2023, reinstated the Rede Cegonha as a model for childbirth, neonatal, and child health care, ensuring access, reception, resolution, and a reduction in maternal and neonatal mortality^(1,5,6)^.

To achieve this, identifying factors that affect the health of women during pregnancy is essential to accelerate efforts aimed at mitigating their potential impact on maternal and fetal health. The health team is responsible for identifying these factors, as well as providing care and specialized referrals in severe cases. Thus, caring for high-risk pregnant women requires professionals who are trained and skilled in managing critical situations^(7)^.

The management of high-risk prenatal care must be carried out by an interprofessional team. In specialized outpatient care, the team should consist, at a minimum, of an obstetrician, nurse or midwife, psychologist, social worker, physical therapist, and nutritionist. The care is continuous and characterized by cycles of individualized services organized into a single care plan^(1)^.

In this context, the use of protocols is a necessary practice to guide interprofessional activities within a health service and to ensure the quality of care processes. Care should be based on scientific evidence to achieve better outcomes; furthermore, it can provide standardized care for high-risk pregnant women, improve the care provided by basic health network professionals, and implement humanized multiprofessional care tailored to each patient’s profile and needs^(4,8,9)^.

The development of protocols must follow methodological steps, including a key component for their legitimacy and credibility: validation. It is important to highlight that, although technical manuals aimed at monitoring pregnant women already exist in the literature, a search for articles was conducted between January and February 2022 by cross-referencing controlled and uncontrolled descriptors in the databases *Literatura Latino-Americana e do Caribe em Ciências da Saúde* (LILACS), *Base de Dados em Enfermagem* (BDENF), Scopus, Web of Science, Cumulative Index to Nursing and Allied Health Literature (CINAHL), and PubMed, totaling 19 articles. However, none of these addressed the development and validation of protocols for interdisciplinary action in high-risk prenatal care. This highlights the necessity and originality of this study.

In light of the above, the following research question was posed: “Is the interprofessional protocol for high-risk prenatal care valid in terms of content?”.

## OBJECTIVES

To develop and validate the content of an interprofessional care protocol for high-risk prenatal care.

## METHODS

### Ethical aspects

This study was approved by the Research Ethics Committee of the Federal University of Piauí. The expert judges who participated in this study were informed about the research objectives and the nature of data collection and signed the Informed Consent Form (ICF).

### Study design and period

This is a methodological study with a quantitative approach, conducted between June 2022 and February 2023. Content validation consists of determining the representativeness of the items that express the content, based on the judgment of specialists in a specific field^(10)^. In this study, two phases were carried out. The first was the development of the protocol using the Convergent Care Research (CCR) methodology^(11)^. The second phase was the content validation, carried out by expert judges^(12)^. The SQUIRE (Standards for QUality Improvement Reporting Excellence) 2.0 instrument from the EQUATOR network was used to guide the methodology.

### Study protocol

The protocol validated in this study is part of the larger research project entitled “Development of an Interprofessional Care Protocol for Pregnant Women at a Maternal and Child Care Center”. The protocol was developed based on the CCR methodology, implemented in four phases: conception, instrumentation, exploration, and analysis^(11)^.

The first phase — conception — involved selecting the topic, defining the guiding question, establishing the research objectives, reviewing the literature, and developing concepts and assumptions. This phase included visiting the study site, conducting literature research on the topic, verifying the existence of interprofessional protocols for specialized care of high-risk pregnant women, and obtaining authorization for implementation at the study location^(11)^.

The second phase — instrumentation — involved the development of methodological procedures, including the selection of the research site, participants, and techniques for data collection and analysis^(11)^. The study was conducted at the Maternal and Child Care Center (CAEMI in Portuguese), a reference unit for high-risk prenatal care located in the municipality of Timon, Maranhão. Seven professionals participated in the study: three nurses, one obstetrician, one social worker, one nutritionist, and one psychologist. To achieve the objectives proposed in the CCR methodology, the following data collection techniques were chosen: participant observation, which allowed the researcher to immerse themselves in practice, and thematic workshops.

The third phase — exploration — corresponds to the collection and recording of data, aimed at obtaining information to produce scientific constructions in research activities and to improve the quality of care provided by the nursing team^(11)^. During participant observation, the Observation Guide was used. This allowed for the observation of the flow of pregnant women seen, the physical structure of the environment, the availability of professionals, the waiting time, and the consultations conducted by the professionals.

Thematic workshops were conducted in four distinct sessions, with the aim of providing a space for discussion among professionals and encouraging participatory dialogue regarding the systematization of care through the protocol. In this regard, the first workshop consisted of presenting the project and discussing the need for systematization; the second facilitated dialogue about the reality of the service; the third focused on analyzing the strengths and weaknesses of the service; and the fourth involved presenting the protocol to the professionals, followed by the necessary changes and adjustments.

In the analysis phase of the CCR, the apprehension process begins with data collection, organization, and coding of the information. The synthesis process involves subjectively examining the data and making associations and variations of the information gathered during the apprehension process. The theorization process involves uncovering the values embedded in the information gathered during the synthesis process^(11)^.

The protocol’s content was organized into the following areas: high-risk prenatal care at CAEMI; patient intake for pregnant women; flowchart of the care process; specific responsibilities of the interprofessional team; collective responsibilities of the interprofessional team; the care pathway for high-risk pregnant women within the healthcare network; and attributes for interprofessional collaborative practice.

Content validation of the protocol was conducted by consensus among the judges^(12)^. Initially, an invitation letter was sent via email and the messaging app WhatsApp® to the selected judges, containing the study objectives, instructions for completing the questionnaire, the protocol to be evaluated, and the link to access the electronic questionnaire (Google Forms), with immediate access to the ICF, which was mandatory for proceeding to the following pages.

The specialists were given a fifteen-day deadline to respond to the questionnaire, with validation being conducted only once by each professional. This phase of the research took place from January to February 2023. For the validation of the protocol, the judges responded to an instrument adapted from Pasquali^(13)^, made available via the Google Forms platform. The tool was used to facilitate the research process as well as for evaluation. The instrument was divided into two sections: the first focused on characterizing the judges, and the second on the variables related to the aspects to be evaluated in the protocol^(13)^.

Data collection was carried out through a multithemed original questionnaire containing 21 semi-structured questions, divided into two parts. The first part generated 10 questions to characterize the sample of judges, covering gender, age, state of residence, city of residence, field of study, degree level, years of professional experience, years of practice with high-risk pregnancies, years of research experience, and the region of Brazil where they work. The second part, consisting of nine questions, focused on the content validation of the protocol.

The judges analyzed the protocol considering^(13)^ its scope (whether the protocol represents the essential behaviors for task execution without omitting any important steps); clarity (whether the protocol is understandable to the lowest level of the target population, using short, simple, and unambiguous sentences); coherence (whether the protocol was formulated in a way that does not appear ridiculous, inappropriate, or childish); and the criticality of the items (whether the protocol addresses essential/ important steps for the success of the task).

Additionally, the elements of objectivity (whether the protocol guides desirable behaviors, allowing only one idea or action); scientific writing (whether the protocol uses language appropriate to the attribute and the professional level of the intended users); relevance (whether the protocol has a defined attribute and contains sentences that cover the same attribute without suggesting different attributes); sequence (whether the protocol has a defined position in the attribute’s continuum); and uniqueness (whether the protocol is distinct and unique in its presentation) were also analyzed^(13)^.

Each aspect of the protocol was evaluated using a Likert scale, with the following options: 1 - not relevant; 2 - slightly relevant; 3 - indifferent; 4 - relevant; and 5 - very relevant. The judges also provided written feedback on the proposed content through the “other” option available on the form. After this phase, the suggestions resulting from the validation process were sent to the authors to inform the final version of the protocol.

### Sample, Inclusion, and Exclusion Criteria

The validation of the protocol was carried out by 11 judges with experience in teaching and clinical practice. The number of judges was determined based on the need for six to twenty specialists to form the committee of judges^(12)^, and their identification and selection followed Jasper’s criteria^(14)^.

Initially, the identification of the first judges took place through the *Lattes* Platform (http://lattes.cnpq.br/), part of the portal of the National Council for Scientific and Technological Development (CNPq). The search for teaching judges was conducted intentionally and randomly, using advanced textual search by subject, with keywords such as high-risk prenatal care, psychology, nutrition, social work, medicine, nursing, and physical therapy, among the profiles of Brazilian PhD holders. A total of 357 curricula were found, and the full abstracts were analyzed, excluding those that did not fit the profile for this study. Invitation letters were sent via email, through the *Lattes* platform, to 88 curricula that met the inclusion criteria. Of these, only four responded with interest in participating within the given timeframe; however, only one completed the questionnaire.

The search for clinical judges followed the inclusion criteria of having more than two years of experience in high-risk pregnancy care and/or high-risk prenatal care. This process was conducted by analyzing judges from the six professional areas covered by the interprofessional protocol (nurses, doctors, physical therapists, social workers, nutritionists, and psychologists). The selection of judges utilized the snowball sampling technique, a convenience sampling method in which the initial participants are asked to refer others who meet the eligibility criteria^(10)^. All those included through this sampling technique responded to the invitation via WhatsApp®, totaling 10 clinical professionals. Thus, out of all the professionals invited to participate in the study, 11 returned the fully completed and evaluated protocol: 10 through the snowball technique and one selected via the *Lattes* platform.

### Analysis of results and statistics

The data collected were exported from Google Forms directly into a Microsoft Excel® 2016 spreadsheet and subsequently analyzed using the Statistical Package for the Social Sciences® (SPSS) version 26. To assess the reliability of the instrument, Cronbach’s alpha coefficient^(15)^ was used, with an acceptable range of 0.70 to 0.90.

The Content Validity Index (CVI) was used to measure the level of agreement among the specialists, considering a CVI ≥ 0.80 as valid. This measurement determines whether the items listed in the protocol are appropriate in terms of content^(16)^. For the CVI calculation, the two highest scores (4 - relevant and 5 - very relevant) were used, divided by the total number of responding judges (CVI = number of responses of 4 or 5 divided by the total number of responses), excluding values of one to three.

## RESULTS

### Main Components of the Protocol

The protocol covered seven main themes: high-risk prenatal care at CAEMI; patient intake for pregnant women; flowchart of the care process; specific responsibilities of the interprofessional team; collective responsibilities of the interprofessional team; the care pathway for high-risk pregnant women within the healthcare network; and attributes for interprofessional collaborative practice.

The first theme, high-risk prenatal care at CAEMI, addressed introductory aspects related to the care provided by the service, the objectives of this care, and how referrals are made to the service. In the theme patient intake for pregnant women, the intake practices carried out by the professionals were defined, including qualified listening and satisfaction surveys through the “*Olá, tudo bem*” project, which initially welcomes pregnant women and informs them about CAEMI’s operations, and the “*Maternando*” project, which guides pregnant women on topics related to pregnancy.

Regarding the theme flowchart of the care process at CAEMI, the service hours and the required documents for referring pregnant women to the high-risk service were described. This theme detailed, through a flowchart, the care process for first-time visitors to the service and for those returning for follow-up consultations.

In the theme specific responsibilities of the interprofessional team, a table was provided listing the responsibilities of each professional involved in the care process: support nurse, obstetric nurse, obstetrician, psychologist, nutritionist, physical therapist, and social worker. Subsequently, the collective responsibilities of the interprofessional team were presented.

The theme care pathway for high-risk pregnant women within the healthcare network detailed the network’s care points at all levels of complexity, based on stratification for high-risk prenatal care. Additionally, the care pathway included instructions for urgent cases and obstetric emergencies that may occur during the care process.

The final theme of the protocol, attributes for interprofessional collaborative practice, covered the necessary attributes for collaborative practice, such as team functioning, communication, role clarification, collaborative leadership, conflict resolution, and patient-centered care.

### Characterization of the judges

The 11 judges who participated in the study were predominantly female (81.8%), with an average age of 36 years, ranging from 28 to 43 years. The majority were nurses (36.4%), with 63.6% holding a *lato sensu* specialization and 63.6% having more than 10 years of professional experience, while 36.4% had no research background. Following Jasper’s criteria^(14)^, all judges met at least two of the requirements (as shown in Table 1), and four judges (36.4%) met three criteria, corresponding to areas of interest such as maternal health, gynecology and obstetrics, and the pregnancy-puerperal cycle.

**Table 1:** Characterization of the participating judges according to Jasper, Teresina, Piauí, Brazil, 2023

Classification criteria for judges	N	%
Possess skill/specialized knowledge that makes the professional an authority on the subject.	11	100.0
Possess skill/knowledge acquired through experience.	11	100.0
Possess special skill in a specific type of study.	7	63.6
Possess approval in a specific test to identify experts.	0	0.0
Possess a high ranking assigned by an authority.	0	0.0

*N – 11 (total number of specialists). % – percentage of the total number.*

### Content validation process of the protocol by the judge

To assess the consistency of the questionnaire used by the judges for protocol validation, Cronbach’s alpha coefficient was calculated. The consistency validation reached a score of 0.889 across the nine items analyzed in the protocol, demonstrating the reliability of the responses provided by the judges.

Regarding scope, 63.6% of the judges considered it relevant, and 36.4% considered it very relevant. In terms of clarity, 45.5% of the judges rated it as relevant, while 54.5% rated it as very relevant. For coherence, four judges (36.4%) marked it as relevant, and seven (63.6%) marked it as very relevant. As for the criticality of the items, three judges (27.3%) considered it relevant, while the majority, eight (72.7%), considered it very relevant.

For objectivity, six judges (54.5%) classified it as relevant, and five (45.5%) classified it as very relevant. Regarding scientific writing, one judge classified it as indifferent (9.1%), five judges (45.5%) classified it as relevant, and five (45.5%) classified it as very relevant. When evaluating relevance, seven judges (63.6%) considered it relevant, while four (36.4%) considered it very relevant. Regarding sequence, seven judges (63.6%) classified it as relevant, and four (36.4%) considered it very relevant. For up-to-dateness, five judges (45.5%) classified it as relevant, and six (54.5%) considered it very relevant.

The overall CVI was 0.990. Of the nine criteria evaluated according to Pasquali^(13)^, eight were validated with a CVI of 1.00, and one was validated with a CVI of 0.91. However, this item was not excluded, as the specialists deemed it appropriate, achieving a satisfactory CVI in the content validation stage (Table 2). The majority of judges did not make additional comments or suggestions; however, one judge pointed out some aspects that needed revision, such as typographical and verbal agreement errors.

**Table 2:** Analysis of internal consistency validation per item and Content Validity Index of expert judges for the protocol, according to Pasquali’s elements, Teresina, Piauí, Brazil, 2023

Elements	1 – Not relevant n (%)	2 - Slightly relevant n (%)	3 - Indifferent n (%)	4 - Relevant n (%)	5 - Very relevant n (%)	CVI	Alpha
Scope	0(0.0)	0(0.0)	0(0.0)	7(63.6)	4(36.4)	1.00	0.880
Clarity	0(0.0)	0(0.0)	0(0.0)	5(45.5)	6(54.5)	1.00	0.864
Coherence	0(0.0)	0(0.0)	0(0.0)	4(36.4)	7(63.6)	1.00	0.891
Criticality of the items	0(0.0)	0(0.0)	0(0.0)	3(27.3)	8(72.7)	1.00	0.877
Objectivity	0(0.0)	0(0.0)	0(0.0)	6(54.5)	5(45.5)	1.00	0.874
Scientific writing	0(0.0)	0(0.0)	1(9.1)	5(45.5)	5(45.5)	0.91	0.876
Relevance	0(0.0)	0(0.0)	0(0.0)	7(63.6)	4(36.4)	1.00	0.857
Sequence	0(0.0)	0(0.0)	0(0.0)	7(63.6)	4(36.4)	1.00	0.857
Up-to-dateness	0(0.0)	0(0.0)	0(0.0)	5(45.5)	6(54.5)	1.00	0.891
General Content Validity Index						0.99	

## DISCUSSION

The development of this study is notable for being a pioneer in the creation and validation of an interdisciplinary protocol aimed at providing care to high-risk pregnant women. Several advantages have been highlighted in the literature regarding the introduction of care protocols to guide healthcare teams, such as the dissemination of safe practices for both users and professionals, the reduction of variability in the execution of care actions, improved decision-making by professionals, the spread of knowledge within the team, as well as enhanced communication and care coordination^(15,16)^.

For work organization, the use of care protocols is necessary as an important management tool, and their application is characteristic of healthcare institutions that prioritize service quality and ensure the safety of both specialists and users. Studies indicate that the adoption of protocols provides support for the organization and management of work processes^(16,17)^.

The interdisciplinary team plays a crucial role in managing high-risk prenatal care and validating protocols, involving these professionals in both the development and content validation processes, which allows for care that is closer to their reality. Research shows that the interdisciplinary participation of specialists in instrument validation is essential to avoid inaccurate results or biased measures that could lead to incorrect conclusions, while incorporating diverse perspectives, cultures, and scientific knowledge to create an appropriate product^(18,19)^.

The validation process of protocols is critical to ensuring the safe use of instruments in clinical practice, as it assesses the proposed objectives and measures what it is intended to evaluate in an appropriate and reliable manner. Validity can be assessed by different methods, depending on the research objective, and can be content, construct, or criterion-related validity^(1,20)^.

It is worth mentioning that this selection process was also carried out in a previous study^(21)^. All the judges in that study met the minimum requirements, and the selection of qualified judges provided a better evaluation of the instrument, ensuring good content validation.

Regarding the expertise of the judges in this study, characterized by their knowledge and skills acquired as healthcare professionals, clinical experience, and their ability to stay up-to-date and critically analyze scientific literature, it is considered essential in validation studies to achieve 100% participation. It is believed that the greater the expertise, the greater the ability to use scientific evidence, involve patients in clinical decision-making, respect their preferences and values, and adjust actions to local conditions^(22)^.

The predominance of female judges observed in this study reflects the changes brought about by women’s increasing participation in the labor market and research. A systematic review indicated that more women are gaining space in the scientific field, although they still face difficulties in career permanence and advancement, even though they are, in many cases, more qualified than men^(23)^.

Regarding professional qualifications, there was a notable participation of PhDs and Masters in this validation study, which contributed to its enhancement. In this regard, researchers report that Masters and PhDs are primarily responsible for promoting evidence-based practices^(22)^.

In terms of the judges’ role in care, a literature review pointed out that professional experience and qualifications in the field are the most considered criteria for selecting specialists in validation processes^(20)^, as was the case in this study. Another study adds that the involvement of experienced professionals in both research and care is relevant for construct validation^(24)^, as seen in this study, which validated a care protocol for high-risk pregnant women.

In health research, content validation is widely used, consisting of two phases: the development of instruments and evaluation by specialists (judges) to determine whether the content is appropriate and accurate in relation to the proposed objective^(24,25)^. One study highlights that the participation of national judges in the content validation process aims to incorporate different experiences and contexts, justifying the choice of Brazilian judges in this study^(26)^.

Considering the need to obtain an instrument that accurately reflects reality, Cronbach’s alpha coefficient was used, which is widely employed in content validation studies to calculate the average correlations between the items comprising the instrument^(24,25,26)^. If the average correlation between items is low, the Cronbach’s alpha value will also be low. If the correlations are high, there is evidence that the items measure the same construct, fulfilling the reliability assessment^(27)^.

It is worth emphasizing that the use of a validated protocol enhances interdisciplinary care, supports the transition of care in managing the assistance network for the target population, and contributes to high-quality specialized care^(28)^.

The CVI has been the most widely used quantitative method to analyze the level of agreement among judges during the instrument validity assessment process^(20,26,29)^. In this study, all the items analyzed were validated, with full consensus among the experts and an excellent global CVI, with the content being validated with at least 90% agreement among the expert judges. It is concluded that content validation of a care protocol allows for the achievement of scientific credibility in care practices.

Among the nine items proposed by Pasquali^(13)^, the items of up-to-dateness and coherence stood out in this study, with a CVI of 1.00 when analyzed individually in all domains. A recent study pointed out that, despite the use of different methods, a review of the scientific literature allows the best and most up-to-date available evidence to be gathered to identify effective healthcare practices^(25)^. These findings may have influenced the high CVI and Cronbach’s alpha scores achieved across all domains when evaluated individually.

Other items highly valued by the judges concerned scope, clarity, criticality of the items, objectivity, and scientific writing. The scientific writing of care instruments is a crucial step, as every care instrument or protocol must be written to avoid any ambiguity, misunderstanding, vagueness, or subjectivity^(22)^.

Relevance and sequence were also validated with a good CVI, and Cronbach’s alpha reached 0.85. It is essential that the content focuses on the proposed theme, with a logical sequence of ideas, moving from more general to specific topics^(15)^.

The use of Google Forms as a tool presented several advantages, but the main disadvantages included questionnaire design, participant dropout, and the long execution time. Several studies^(30,31)^ have used this tool for data collection, supporting the broader reach of professionals in this research. Additionally, it was possible to standardize the responses so that similar answers were considered equivalent during the analysis^(32)^. Thus, it is important to standardize the questions, the participant group, and the environment in which the questionnaire is applied to avoid influences on the responses.

### Study limitations

The difficulty in engaging the convenience sample of judges was a noted limitation. A low response and return rate was observed, consistent with the disadvantages often associated with online data collection. Additionally, the scarcity of validation studies for technologies such as care protocols for high-risk pregnant women made it challenging to compare and discuss the results.

### Contributions to the Field of Nursing and Public Health

The dissemination of this study helps fill a perceived gap. It is expected that this research will contribute to initiatives aimed at expanding possibilities for safe and comprehensive care by professionals involved in high-risk pregnancy care through the use of new technologies, such as care protocols.

## CONCLUSIONS

The interprofessional care protocol for specialized prenatal care covered topics related to high-risk prenatal care at CAEMI, patient intake for pregnant women, the flowchart of the care process, specific responsibilities of the interprofessional team, collective responsibilities of the interprofessional team, the care pathway for high-risk pregnant women within the healthcare network, and the attributes for interprofessional collaborative practice.

The protocol was found to be valid in terms of content, with a high level of recommendation from the expert judges for use by an interdisciplinary team in caring for high-risk pregnant women. The data reinforce that the validated material will contribute to clinical practice by helping to systematize, organize, and structure care, promoting improvements in the quality of high-risk prenatal care and guiding interdisciplinary teams.

As this is a pioneering study in the development and validation of a care protocol for high-risk pregnant women, it will bring benefits to the development of new validated care technologies and their wide dissemination within the scientific community. Future studies are recommended to apply and evaluate the protocol.

## References

[1] Ministério da Saúde (BR) (2019). Nota Técnica para Organização da Rede de Atenção à Saúde com Foco na Atenção Primária à Saúde e na Atenção Ambulatorial Especializada: Saúde da Mulher na Gestação, Parto e Puerpério [Internet].

[2] Organização Mundial da Saúde (OMS) (2019). Maternal mortality[Internet].

[3] Programa das Nações Unidas para o Desenvolvimento (PNUD) (2015). Acompanhando a agenda 2030 para o desenvolvimento sustentável: subsídios iniciais do Sistema das Nações Unidas no Brasil sobre a identificação de indicadores nacionais referentes aos objetivos de desenvolvimento sustentável [Internet].

[4] Ministério da Saúde (BR) (2022). Manual de gestação de alto risco. Secretaria de Atenção Primária à Saúde. Departamento de Ações Programáticas [Internet].

[5] Ministério da Saúde (BR) (2011). Portaria nº 1.459, de 24 de junho de 2011. Institui, no âmbito do Sistema Único de Saúde SUS: a Rede Cegonha [Internet].

[6] Ministério da Saúde (BR) (2023). Portaria GM/MS nº 13, de 13 de janeiro de 2023. Revoga Portarias que especifica e dá outras providências[Internet].

[7] Alves TO, Nunes RLN, Sena LHA, Alves FG, Souza AGS, Salviano AGS (2021). High risk pregnancy: epidemiology and care, a literature review. Braz J Health Rev.

[8] Azevedo CS, Miranda L, Sá MC, Grabois V, Matta G, Cunha M. (2018). [Between protocols and subject: quality of hospital care in a hematology service]. Cad Saúde Pública.

[9] Evaldt RFS, Hartmann AE, Santos BZ. (2022). Protocolo para o pré-natal de alto risco [Internet].

[10] Polit DF, Beck CT. (2019). Fundamentos de pesquisa em enfermagem: avaliação de evidências para a prática de enfermagem.

[11] Trentini M, Paim L, Silva DGV. (2014). Pesquisa convergente assistencial: delineamento provocador de mudanças nas práticas de saúde.

[12] Alexandre NM, Coluci MZ. (2011). [Content validity in the development and adaptation processes of measurement instruments]. Ciênc Saúde Coletiva.

[13] Pasquali L. (2010). Instrumentação psicológica-fundamentos e práticas.

[14] Jasper MA. (1994). Expert: a discussion of the implications of the concept as used in nursing. J Adv Nurs.

[15] Cronbach LJ. (1951). Coefficient alpha and the internal structure of test. Psychometrika.

[16] Krauzer IM, Dall’Agnoll CM, Gelbcke FL, Lorenzini E, Ferraz L. (2018). The construction of assistanceprotocols in nursing work. Rev Min Enferm.

[17] Rigo AP, Levandovski RM. (2021). A practical guide for accessing the medications of the Specialized Component listed in the Clinical Protocols and Therapeutic Guidelines of the Brazilian Ministry of Health. CaEPS.

[18] Leite SS, Áfio ACE, Carvalho LV, Silva JM, Almeida PC, Pagliuca LMF. (2018). Construction and validation of an Educational Content Validation Instrument in Health. Rev Bras Enferm.

[19] Pedrosa KKA, Oliveira SA, Machado RC. (2018). Validation of a care protocol for the septic patient in the Intensive Care Unit. Rev Bras Enferm.

[20] Vieira TW, Sakamoto VTM, Moraes LC, Blatt CR, Caregnato RCA. (2020). Validation methods of nursing protocols: an integrative review. Rev Bras Enferm.

[21] Souto REM. (2022). Construção e validação de um questionário de identificação de violência obstétrica[Dissertação] [Internet].

[22] Pimenta CAM. (2017). Guia para a implementação de protocolos assistenciais de enfermagem: integrando protocolos, prática baseada em evidência e classificações de enfermagem [Internet].

[23] Ibarra ACR, Ramos NB, Oliveira MZ. (2021). Desafios das mulheres na carreira científica no Brasil: uma revisão sistemática. Rev Bras Orientac Prof [Internet].

[24] Gomes ATL, Alves KYA, Bezerril MS, Rodrigues CCFM, Ferreira MA, Santos VEP. (2018). Validation of graphic protocols to evaluate the safety of polytrauma patients. Acta Paul Enferm.

[25] Catunda HLO, Bernardo EBR, Vasconcellos CTM, Moura ERF, Pinheiro AKB, Aquino PS. (2017). Methodological approach in nursing research for constructing and validating protocols. Texto Contexto Enferm.

[26] Rodrigues JAP, Lacerda MR, Galvão CM, Cubas MR, Kalinke LP, Gomes IM (2022). Content validation of nursing care protocol in pediatric hematopoietic stem cells post-transplantation. Res, Soc Dev.

[27] Souza AC, Alexandre NMC, Guirardello EB. (2017). Psychometric properties in instruments evaluation of reliability and validity. Epidemiol Serv Saúde.

[28] Santos NOD, Predebon ML, Bierhals CCBK, Day CB, Machado DO, Paskulin LMG. (2020). Development and validation a nursing care protocol with educational interventions for family caregivers of elderly people after stroke. Rev Bras Enferm.

[29] Ferreira CIV, Simões IMH. (2019). Validation of urinary retention evaluation nursing protocol and nursing diagnosis in adults. Rev Enf Ref.

[30] Mota JS. (2019). Use of Google Forms in academic research. Rev Human Inov [Internet].

[31] Portela MG, Mello AL, Carmo DRP, Freitas EO, Siqueira DF. (2021). Online data collection in quantitative research: experience with university health students. RECIMA21.

[32] Neves C, Augusto C, Terra AL. (2020). Online surveys: comparative tool analysis for the creation and administration of e-surveys. AtoZ.

