# Supplementary material for: Development and validation of an interprofessional protocol for high-risk prenatal care
Source: Rev Bras Enferm. 2025 Mar 10;78(1):e20230197. doi: 10.1590/0034-7167-2023-0197 (PMC11893058; doi:10.1590/0034-7167-2023-0197)
Supplement: Translation

# Translation: Construção e validação de protocolo interprofissional para o cuidado pré-natal na gravidez de alto risco

**DOI:** 10.1590/0034-7167-2023-0197pt

**Authors:** Daniella Carvalho Araújo, Láisa Rebecca Sousa Carvalho, Leyla Gerlane de Oliveira Adriano, Ravena de Sousa Alencar Ferreira, Girzia Sammya Tajra Rocha, Priscila de Souza Aquino, Victórya Suéllen Maciel Abreu, Herla Maria Furtado Jorge

**Affiliations:** I Universidade Federal do Piauí. Teresina, Piauí, Brasil; II Universidade Federal do Ceará. Fortaleza, Ceará, Brasil

**Keywords:** Estudo de Validação, Cuidado Pré-Natal, Gravidez de Alto Risco, Protocolo, Equipe Interdisciplinar de Saúde

## Abstract

**Objetivos:**

construir e validar o conteúdo de um protocolo assistencial interprofissional para o cuidado pré-natal de alto risco.

**Métodos:**

estudo metodológico com abordagem quantitativa, realizado de junho de 2022 a fevereiro de 2023. A primeira fase do estudo consistiu na elaboração do protocolo por meio da Pesquisa Convergente Assistencial, e, na segunda, realizou-se a validação de conteúdo por meio da avaliação do protocolo por 11 juízes especialistas. Utilizou-se o Índice de Validade de Conteúdo (IVC) ≥ 0,80 para análise do grau de concordância entre os juízes, e o alfa de Cronbach para a confiabilidade do instrumento.

**Resultados:**

atingiu-se a validação de conteúdo do protocolo com IVC geral de 0,990 e validação interna com alfa de Cronbach de 0,889.

**Conclusões:**

o protocolo é válido quanto ao conteúdo, com alto grau de recomendação pelos juízes, o que promoverá melhorias na qualidade da assistência pré-natal de alto risco.

## INTRODUÇÃO

A atenção ao pré-natal de alto risco integra características e necessidades específicas, sobretudo no que diz respeito às orientações sobre o tipo de parto mais seguro em cada situação^(1)^. Aproximadamente 830 mulheres morrem todos os dias por complicações relacionadas à gravidez ou ao parto no mundo. Em 2017, houve cerca de 295 mil casos de mortes de mulheres durante e após a gravidez e o parto, principalmente em ambientes com recursos escassos, sendo casos considerados evitáveis. A atenção à saúde materna ganhou destaque no mundo como um dos Objetivos de Desenvolvimento do Milênio, estabelecidos pela Organização Mundial da Saúde no ano 2000. Este marco reconfigura o quadro de preocupações mundiais, com a estimativa de redução da taxa de mortalidade materna global para menos de 70 mortes por 100.000 nascidos vivos^(2)^.

No Brasil, políticas públicas foram implementadas desde o início deste milênio, como o desenvolvimento do Programa de Humanização no Pré-natal e Nascimento, que visa melhorar a qualidade obstétrica e reduzir as mortalidades materna e perinatal^(3,4)^. A Portaria GM/MS nº13, de janeiro de 2023, do Ministério da Saúde, retomou a Rede Cegonha como modelo de atenção ao parto, nascimento e à saúde da criança, garantindo acesso, acolhimento, resolutividade e redução da mortalidade materna e neonatal^(1,5,6)^.

Para isso, a identificação de fatores que interferem na situação de saúde da mulher durante a gestação é fundamental, de forma a acelerar a atuação destinada a modificar e minimizar o possível impacto desses fatores na saúde do binômio materno-fetal. É responsabilidade da equipe de saúde essa identificação, bem como o atendimento e o encaminhamento especializado nos casos graves. Assim, a assistência à gestante de alto risco necessita de capacitação e aptidão dos profissionais no manejo de situações de alerta^(7)^.

O manejo do pré-natal de alto risco deve ser realizado por uma equipe interprofissional. Na atenção ambulatorial especializada, a equipe deve ser composta, no mínimo, por médico obstetra, enfermeiro ou obstetriz, psicólogo, assistente social, fisioterapeuta e nutricionista. A assistência é contínua, caracterizada por ciclos de atendimentos individuais sistematizados em um único plano de cuidados^(1)^.

Nesse contexto, o uso de protocolos constitui uma prática necessária para nortear as atividades interprofissionais dentro de um serviço de saúde e garantir a qualidade dos processos assistenciais. A assistência deve ser ancorada em evidências científicas para alcançar melhores resultados; além disso, pode proporcionar um cuidado padronizado para gestantes de alto risco, qualificar o atendimento dos profissionais da rede básica e implantar a assistência humanizada multiprofissional, conforme o perfil e a necessidade de cada paciente^(4,8,9)^.

No desenvolvimento de protocolos, devem-se seguir etapas metodológicas que incluem um importante componente para sua legitimidade e credibilidade, que é a validação. É importante destacar que, embora já existam na literatura manuais técnicos direcionados ao acompanhamento de gestantes, foi realizada, entre janeiro e fevereiro de 2022, uma busca de artigos por meio do cruzamento de descritores controlados e não controlados nas bases de dados Literatura Latino-Americana e do Caribe em Ciências da Saúde (LILACS), Base de Dados em Enfermagem (BDENF), Scopus, Web of Science, *Cumulative Index to Nursing and Allied Health Literature* (CINAHL) e PubMed, totalizando 19 artigos, porém, nenhum contemplou a construção e validação de protocolos sobre a atuação interdisciplinar no pré-natal de alto risco. Isso demonstra a necessidade e originalidade deste estudo.

Diante do exposto, elencou-se a seguinte questão de pesquisa: “O protocolo interprofissional de cuidado pré-natal de alto risco é válido quanto ao conteúdo?”.

## OBJETIVOS

Construir e validar o conteúdo de um protocolo assistencial interprofissional para o cuidado pré-natal de alto risco.

## MÉTODOS

### Aspectos éticos

Este estudo foi aprovado pelo Comitê de Ética em Pesquisa da Universidade Federal do Piauí. Os juízes especialistas que participaram deste estudo foram esclarecidos quanto ao objetivo da investigação e à natureza da coleta de dados, e assinaram o Termo de Consentimento Livre e Esclarecido (TCLE).

### Desenho e período do estudo

Trata-se de um estudo metodológico com abordagem quantitativa, realizado no período de junho de 2022 a fevereiro de 2023. A validação de conteúdo consiste na determinação da representatividade dos itens que expressam um conteúdo, baseada no julgamento de especialistas em uma área específica^(10)^. Neste estudo, foram realizadas duas fases. A primeira foi a elaboração do protocolo por meio da Pesquisa Convergente Assistencial (PCA)^(11)^. A segunda fase foi a validação de conteúdo, por meio da avaliação do protocolo por juízes^(12)^. Utilizou-se o instrumento SQUIRE (*Standards for QUality Improvement Reporting Excellence*) 2.0, da rede EQUATOR, para nortear a metodologia.

### Protocolo do estudo

O protocolo validado neste estudo integra o macroprojeto de pesquisa intitulado “Desenvolvimento de protocolo de cuidado interprofissional às gestantes de um centro de atenção mater-no-infantil”. O protocolo foi construído com base no referencial metodológico da PCA, implementada em quatro fases: concepção, instrumentação, perscrutação e análise^(11)^.

A primeira fase — concepção — representa a escolha do tema, o direcionamento da questão-guia, o estabelecimento dos objetivos da pesquisa, a revisão da literatura, a elaboração de conceitos e pressupostos. Essa fase compreendeu a visita ao cenário do estudo, pesquisa na literatura sobre o tema, verificação da existência de protocolos interprofissionais na atenção especializada à gestante de alto risco, e a possibilidade e autorização para implantação no local do estudo^(11)^.

A segunda fase — instrumentação — consiste na elaboração dos procedimentos metodológicos, incluindo a escolha do espaço da pesquisa, dos participantes e da técnica para obtenção e análise das informações^(11)^. O estudo foi realizado no Centro de Atenção Materno-Infantil (CAEMI), unidade de referência para a atenção pré-natal de alto risco, localizado no município de Timon, Maranhão. Participaram do estudo sete profissionais: três enfermeiros, um médico obstetra, um assistente social, um nutricionista e uma psicóloga. Para atingir os objetivos propostos na metodologia da PCA, foram escolhidas as seguintes técnicas de coleta de dados: observação participante, que permitiu a imersão do pesquisador na prática, e a realização de oficinas temáticas.

A terceira fase — perscrutação — corresponde à coleta e ao registro dos dados, destinados a obter informações para produzir construções científicas nas atividades de pesquisa e favorecer o aperfeiçoamento do cuidado prestado pela equipe de enfermagem^(11)^. Na observação participante, utilizou-se o Roteiro de Observação. Dessa forma, foi possível observar o fluxo das gestantes atendidas, a estrutura física do ambiente, a disponibilidade dos profissionais, o tempo de espera e as consultas realizadas pelos profissionais.

As oficinas temáticas foram realizadas em quatro momentos distintos e tinham como finalidade proporcionar um espaço de discussão entre os profissionais e favorecer o diálogo participativo acerca da sistematização do cuidado por meio do protocolo. Nesse sentido, a primeira oficina consistiu na apresentação do projeto e discussão sobre a necessidade de sistematização; a segunda possibilitou o diálogo sobre a realidade do serviço; a terceira consistiu na análise das potencialidades e fragilidades do serviço; e a quarta consistiu na apresentação do protocolo para os profissionais, seguida pelas mudanças e alterações necessárias.

Na fase de análise da PCA, o processo de apreensão inicia com a coleta de dados, organização e codificação das informações; o processo de síntese consiste em examinar subjetivamente os dados e realizar associações e variações das informações encontradas no processo de apreensão; e o processo de teorização consiste em descobrir os valores contidos nas informações levantadas durante o processo de síntese^(11)^.

O conteúdo do protocolo foi organizado nos seguintes eixos: O pré-natal de alto risco no CAEMI; acolhimento das gestantes; fluxograma do circuito de atendimento; atribuições específicas da equipe interprofissional; atribuições coletivas da equipe interprofissional; fluxo de atendimento da gestante de alto risco na rede de atenção; e atributos para a prática interprofissional colaborativa.

A validação de conteúdo do protocolo foi realizada por meio do consenso entre os juízes^(12)^. Inicialmente, foi enviada uma carta-convite por e-mail e pelo aplicativo de mensagens WhatsApp® para os juízes selecionados, contendo os objetivos do estudo, instruções para preenchimento do questionário, o protocolo a ser avaliado e o link de acesso ao questionário eletrônico (*Google Forms*), com abertura imediata ao TCLE, sendo obrigatória para o acesso às páginas seguintes.

Foi solicitado aos especialistas um prazo de quinze dias para responder ao formulário, sendo a validação realizada apenas uma vez por cada profissional. O período dessa etapa da pesquisa foi de janeiro a fevereiro de 2023. Para a validação do protocolo, os juízes responderam a um instrumento adaptado de Pasquali^(13)^, disponibilizado por meio da plataforma *Google Forms*. A ferramenta foi utilizada para facilitar o processo de pesquisa, bem como para avaliação. Assim, o instrumento foi dividido em duas seções: a primeira referiu-se à caracterização dos juízes, e a segunda às variáveis dos aspectos a serem avaliados no protocolo^(13)^.

A coleta de dados se deu por meio de um questionário multitemático original, contendo 21 perguntas semiestruturadas, segmentadas em duas partes. A primeira parte gerou 10 questões para a caracterização da amostra de juízes, abordando: gênero, idade, estado de residência, cidade de residência, área de formação, grau de formação, tempo de atuação profissional, tempo de prática com gestantes de alto risco, anos de pesquisa e estado do Brasil em que atua. A segunda parte, com nove questões, focou na validação do conteúdo do protocolo.

Os juízes analisaram o protocolo considerando^(13)^ a abrangência (se o protocolo representa os comportamentos essenciais à execução da tarefa, sem omitir nenhum passo importante); clareza (se o protocolo é inteligível para o estrato mais baixo da população-alvo, com uso de frases curtas, expressões simples e inequívocas); coerência (se o protocolo foi formulado de modo que não pareça ridículo, despropositado ou infantil); e criticidade dos itens (se o protocolo aborda passos essenciais/importantes para o sucesso da tarefa).

Foram analisados ainda os elementos de objetividade (se o protocolo orienta a comportamentos desejáveis, permitindo uma só ideia ou atitude); redação científica (se o protocolo possui frases adequadas ao atributo e ao nível de formação do profissional que fará uso do instrumento); relevância (se o protocolo possui o atributo definido e frases que cobrem o mesmo atributo, sem insinuar atributos diferentes do definido); sequência (se o protocolo tem uma posição definida no contínuo do atributo); e unicidade (se o protocolo é distinto, único em sua apresentação)^(13)^.

Cada aspecto do protocolo foi avaliado de acordo com uma escala do tipo Likert, considerando-se: 1 - não relevante; 2 - pouco relevante; 3 - indiferente; 4 - relevante; e 5 - bastante relevante. Os juízes também emitiram opiniões por escrito quanto ao conteúdo proposto, por meio da opção “outros”, disponibilizada pelo formulário. Após esta etapa, as sugestões provenientes do processo de validação foram encaminhadas aos autores para conduzir a versão final do protocolo.

### Amostra, critérios de inclusão e exclusão

A validação do protocolo foi realizada por 11 juízes com experiência nas áreas de docência e assistência. O quantitativo de juízes foi definido considerando-se necessário um número de seis a vinte especialistas para compor o comitê de juízes^(12)^, e sua identificação e seleção seguiram os critérios de Jasper^(14)^.

Inicialmente, a identificação dos primeiros juízes ocorreu por meio da Plataforma *Lattes* (http://lattes.cnpq.br/), do portal do Conselho Nacional de Desenvolvimento Científico e Tecnológico (CNPq). A busca pelos juízes docentes foi feita de forma intencional e aleatória, utilizando a busca textual avançada por assunto, com as palavras-chave: pré-natal de alto risco, psicologia, nutrição, assistente social, medicina, enfermagem e fisioterapia, nas bases de doutores de nacionalidade brasileira. Foram encontrados 357 currículos, nos quais os textos completos dos resumos foram analisados, excluindo-se os que não se enquadravam no perfil para este estudo. A carta-convite foi enviada por e-mail, pela plataforma *Lattes*, para os 88 currículos que contemplavam os critérios de inclusão. Desses, apenas quatro responderam com interesse em participar do estudo em tempo hábil, contudo, apenas um respondeu ao formulário.

A busca pelos juízes assistenciais seguiu o critério de inclusão: experiência de mais de dois anos em atendimento a gestantes de alto risco e/ou pré-natal de alto risco. Esse processo foi conduzido por análise de juízes das seis áreas de abrangência do protocolo interprofissional (enfermeiros, médicos, fisioterapeutas, assistentes sociais, nutricionistas e psicólogos). Para a seleção dos juízes, foi utilizada a técnica em cadeia de referência ou bola de neve, que consiste em uma técnica de amostragem por conveniência, na qual se solicita que os primeiros membros da amostra indiquem outras pessoas que atendam aos critérios de elegibilidade^(10)^. Todos os incluídos por essa amostragem responderam ao convite via aplicativo de mensagens WhatsApp®, totalizando 10 profissionais assistenciais. Logo, de todos os profissionais convidados para o estudo, 11 devolveram o protocolo completamente analisado e avaliado: 10 profissionais pela técnica bola de neve e um profissional selecionado via *Lattes*.

### Análise dos resultados e estatísticas

Os dados coletados foram exportados do *Google Forms* diretamente para uma planilha do *Microsoft Excel*® versão 2016 e, posteriormente, analisados no *Software Statistical Package for the Social Sciences*® (SPSS) versão 26. Para avaliar a confiabilidade do instrumento, foi utilizado o coeficiente alfa de Cronbach^(15)^, que deve ter um valor mínimo de 0,70 e máximo de 0,90.

Utilizou-se o Índice de Validade de Conteúdo (IVC) para medir o nível de concordância entre os especialistas, considerando válido o IVC ≥ 0,80. Essa mensuração determina se os itens elencados no protocolo apresentam adequação do conteúdo^(16)^. Para o cálculo do IVC, adotaram-se as duas pontuações mais elevadas (4 - relevante e 5 - bastante relevante), divididas pelo número total de juízes respondentes (IVC = número de respostas 4 ou 5 dividido pelo total de respostas), excluindo-se os valores de um a três.

## RESULTADOS

### Principais componentes do protocolo

O protocolo contemplou sete temáticas: O pré-natal de alto risco no CAEMI; Acolhimento das gestantes; Fluxograma do circuito de atendimento; Atribuições específicas da equipe interprofissional; Atribuições coletivas da equipe interprofissional; Fluxo de atendimento da gestante de alto risco na rede de atenção; e Atributos para a prática interprofissional colaborativa.

A primeira temática — O pré-natal de alto risco no CAEMI — abordou os aspectos introdutórios relacionados à assistência prestada pelo serviço, o objetivo dessa assistência e como ocorre o encaminhamento para este serviço. Na temática Acolhimento das gestantes, foram definidas as práticas de acolhimento realizadas pelos profissionais na assistência, como: escuta qualificada, pesquisa de satisfação por meio dos projetos “Olá, tudo bem”, que objetiva acolher inicialmente as gestantes e orientá-las quanto ao funcionamento do CAEMI, e o “Maternando”, direcionado para orientar as gestantes sobre temas relacionados à gestação.

Em relação ao Fluxograma do circuito de atendimento no CAEMI, foram descritos os horários de atendimento e os documentos necessários para o referenciamento da gestante para o serviço de alto risco. Nessa temática, foi detalhado, por meio de fluxograma, o circuito de atendimento da gestante que comparece pela primeira vez ao serviço e das que realizarão consultas subsequentes.

Na temática Atribuições específicas da equipe interprofissional, foi inserido um quadro contendo as atribuições de todos os profissionais que compõem o circuito de atendimento: enfermeiro de apoio, enfermeiro obstetra, médico obstetra, psicólogo, nutricionista, fisioterapeuta e assistente social. Em seguida, foram apresentadas as atribuições coletivas da equipe interprofissional.

A temática Fluxo de atendimento da gestante de alto risco na rede de atenção detalhou os pontos de atenção da rede em todos os níveis de complexidade, de acordo com a estratificação para o pré-natal de alto risco. Além disso, o fluxo incluiu o direcionamento para os casos de urgência e as intercorrências obstétricas que possam ocorrer durante o circuito de atendimento.

A última temática do protocolo — Atributos para a prática interprofissional colaborativa — contemplou os atributos necessários para a prática colaborativa, tais como: funcionamento da equipe, comunicação, clarificação de papéis, liderança cola-borativa, resolução de conflitos e cuidado centrado no paciente.

### Caracterização dos juízes

Os 11 juízes que participaram do estudo eram predominantemente do sexo feminino (81,8%), com média de idade de 36 anos, variando de 28 a 43 anos. Prevaleceram juízes enfermeiros (36,4%), com especialização lato sensu (63,6%) e mais de 10 anos de exercício profissional (63,6%), sendo que 36,4% não possuíam trajetória de pesquisa. Seguindo os critérios de Jasper^(14)^, todos os juízes atenderam a no mínimo dois requisitos (como mostra a Tabela 1, e quatro juízes (36,4%) se enquadraram em três requisitos, de acordo com a área de interesse: saúde materna, ginecologia e obstetrícia, e ciclo gravídico-puerperal.

**Tabela 1:** Caracterização dos juízes participantes, segundo Jasper, Teresina, Piauí, Brasil, 2023

Critérios de classificação dos juízes	N	%
Possuir habilidade/ conhecimento especializado que torna o profissional uma autoridade no assunto.	11	100,0
Possuir habilidade/ conhecimento adquirido(s) pela experiência	11	100,0
Possuir habilidade especial em determinado tipo de estudo	7	63,6
Possuir aprovação em um teste específico para identificar experts	0	0,0
Possuir classificação alta atribuída por uma autoridade.	0	0,0

*N – 11 (número total de especialistas). % - porcentagem do número total.*

### Processo de validação do conteúdo do protocolo pelos juízes

Para medir a consistência do questionário utilizado pelos juízes para a validação do protocolo, foi calculado o coeficiente alfa de Cronbach. A validação da consistência atingiu a pontuação de 0,889 entre os nove itens analisados no protocolo, demonstrando a confiabilidade nas respostas ao formulário utilizado pelos juízes.

Quanto à abrangência, os juízes consideraram entre relevante (63,6%) e bastante relevante (36,4%). Em relação à clareza, os juízes pontuaram entre relevante (45,5%) e muito relevante (54,5%). Sobre a coerência, quatro juízes (36,4%) marcaram como relevante e sete (63,6%) marcaram como muito relevante. No tocante à criticidade dos itens, três juízes (27,3%) consideraram relevante e a maioria, oito (72,7%), consideraram muito relevante.

Quanto à objetividade, seis juízes (54,5%) classificaram como relevante e cinco (45,5%) classificaram como muito relevante. Sobre a redação científica, um juiz classificou como indiferente (9,1%), cinco (45,5%) como relevante e cinco (45,5%) como muito relevante. Ao avaliarem a relevância, sete juízes (63,6%) consideraram relevante, enquanto quatro (36,4%) consideraram muito relevante. Sobre a sequência, sete juízes (63,6%) consideraram relevante e quatro (36,4%) consideraram muito relevante. Quanto à atualização, cinco juízes (45,5%) classificaram como relevante e seis (54,5%) como muito relevante.

O IVC geral foi de 0,990. Quanto aos nove critérios avaliados conforme Pasquali^(13)^, oito foram validados com IVC = 1,00, e um foi validado com IVC = 0,91; contudo, não foi excluído, pois os especialistas o julgaram adequado e atingiu um IVC satisfatório na etapa de validação de conteúdo (Tabela 2). A maioria dos juízes não fez considerações ou sugestões adicionais, entretanto, um juiz ressaltou alguns pontos a serem revisados, como erros de digitação e concordância verbal.

**Tabela 2:** Análise de validação da consistência interna por item e Índice de Validação de Conteúdo de juízes especialistas pelo protocolo, segundo os elementos de Pasquali, Teresina, Piauí, Brasil, 2023

Elementos	1 - Não relevante n (%)	2 - Pouco relevante n (%)	3 - Indiferente n (%)	4 - Relevante n (%)	5 - Bastante relevante n (%)	IVC	Alpha
Abrangência	0(0,0)	0(0,0)	0(0,0)	7(63,6)	4(36,4)	1,00	0,880
Clareza	0(0,0)	0(0,0)	0(0,0)	5(45,5)	6(54,5)	1,00	0,864
Coerência	0(0,0)	0(0,0)	0(0,0)	4(36,4)	7(63,6)	1,00	0,891
Criticidade dos itens	0(0,0)	0(0,0)	0(0,0)	3(27,3)	8(72,7)	1,00	0,877
Objetividade	0(0,0)	0(0,0)	0(0,0)	6(54,5)	5(45,5)	1,00	0,874
Redação científica	0(0,0)	0(0,0)	1(9,1)	5(45,5)	5(45,5)	0,91	0,876
Relevância	0(0,0)	0(0,0)	0(0,0)	7(63,6)	4(36,4)	1,00	0,857
Sequência	0(0,0)	0(0,0)	0(0,0)	7(63,6)	4(36,4)	1,00	0,857
Atualização	0(0,0)	0(0,0)	0(0,0)	5(45,5)	6(54,5)	1,00	0,891
Índice de Validação de Conteúdo Geral						0,99	

## DISCUSSÃO

O desenvolvimento deste estudo é destacável por ser pioneiro na construção e validação de um protocolo interdisciplinar voltado aos cuidados assistenciais à gestante de alto risco. Diversas vantagens têm sido apontadas pela literatura em relação à introdução de proto -colos assistenciais para direcionar as práticas de cuidado da equipe de saúde, como: disseminação de práticas seguras para usuários e profissionais, redução da heterogeneidade na condução das ações de cuidado, melhora na tomada de decisões pelos profissionais, disseminação de conhecimento entre a equipe, além de aprimorar a comunicação e a coordenação do cuidado^(15,16)^.

Para a organização do trabalho, faz-se necessário o uso de protocolos assistenciais como um importante instrumento de gerenciamento, sendo sua aplicação característica de instituições de saúde que prezam pela qualidade dos serviços e pela garantia de segurança tanto para especialistas quanto para usuários. Estudos indicam que a adoção de protocolos fornece subsídios à organização e gestão do trabalho^(16,17)^.

A equipe interdisciplinar desempenha um papel essencial no manejo da atenção ao pré-natal de alto risco e na validação de protocolos, trazendo esses profissionais para o processo tanto na construção quanto na validação de conteúdo, o que possibilita uma assistência mais próxima da realidade deles. Pesquisas apontam que a participação interdisciplinar de especialistas na validação de instrumentos é fundamental para evitar resultados imprecisos ou medidas tendenciosas que levem a conclusões equivocadas, com visões, culturas e conhecimento científico que tornam o produto adequado^(18,19)^.

O processo de validação de protocolos é imprescindível para garantir a segurança no uso de instrumentos na prática assistencial, pois analisa os objetivos propostos e mede de forma adequada e fidedigna aquilo que se propõe a avaliar. A validade pode ser analisada por diferentes métodos, dependendo do objetivo da pesquisa, podendo ser de conteúdo, de constructo ou relacionada a um critério^(1,20)^.

Destaca-se que essa forma de seleção também foi realizada em um estudo^(21)^. Todos os juízes desse estudo atenderam ao mínimo de requisitos, e a seleção de juízes qualificados proporcionou uma melhor avaliação do instrumento, garantindo uma boa validação de conteúdo.

Quanto à expertise dos juízes deste estudo, caracterizada pelos conhecimentos e habilidades adquiridos pelos profissionais de saúde, experiência clínica e sua aptidão para atualização e análise crítica da literatura científica, considera-se primordial em estudos de validação atingir 100% dos participantes. Acredita-se que, quanto maior a expertise, maior a capacidade de utilizar evidências científicas, envolver os pacientes na tomada de decisões clínicas, respeitar suas preferências e valores, e adequar as ações às condições locais^(22)^.

A hegemonia do sexo feminino observada entre os juízes deste estudo reflete as mudanças marcadas pela inserção da mulher no mercado de trabalho e na pesquisa. Uma revisão sistemática apontou que mais mulheres estão conquistando espaço no meio científico, embora ainda enfrentem dificuldades de permanência e promoção na carreira, mesmo sendo, em muitos casos, mais qualificadas que os homens^(23)^.

Quanto à qualificação profissional, observou-se uma acentuada participação de doutores e mestres neste estudo de validação, o que colaborou para seu aperfeiçoamento. Nesse sentido, pesquisadores relatam que mestres e doutores são os principais responsáveis por fomentar práticas baseadas em evidências^(22)^.

No que tange à atuação dos juízes na assistência, uma revisão da literatura apontou que a experiência profissional e a titulação na área são os critérios mais considerados para a seleção de especialistas em processos de validação^(20)^, como foi o caso deste estudo. Um outro estudo acrescenta que a participação de profissionais experientes e envolvidos na pesquisa e na assistência é relevante para a validação de constructos^(24)^, como ocorreu neste estudo, que validou um protocolo assistencial para gestantes de alto risco.

Em pesquisas na área da saúde, a validação de conteúdo é amplamente utilizada, consistindo em duas fases: o desenvolvimento de instrumentos e a avaliação por especialistas (juízes) para determinar se o conteúdo está adequado e correto em relação ao objetivo proposto^(24,25)^. Um estudo destaca que a participação de juízes nacionais no processo de validação de conteúdo tem como objetivo abordar diferentes vivências e contextos, o que justifica a escolha de juízes brasileiros neste estudo^(26)^.

Considerando a necessidade de obter um instrumento que retrate a realidade de forma confiável, foi utilizado o coeficiente alfa de Cronbach, amplamente empregado em estudos de validação de conteúdo, para calcular a média das correlações entre os itens que compõem o instrumento^(24,25,26)^. Se a média entre os itens for baixa, o valor do coeficiente alfa de Cronbach também será baixo. Se as correlações forem altas, há evidências de que os itens medem o mesmo constructo, satisfazendo a avaliação da confiabilidade^(27)^.

Vale ressaltar que a utilização de um protocolo validado qualifica o cuidado interdisciplinar, apoia a transição do cuidado no gerenciamento da rede de assistência à população-alvo e contribui para um cuidado especializado de qualidade^(28)^.

O uso do IVC tem sido o método quantitativo mais utilizado para analisar o grau de concordância entre os juízes durante o processo de avaliação da validade de um instrumento^(20,26,29)^. Neste estudo, todos os itens analisados foram validados, com consenso total entre os peritos e um excelente IVC global, sendo o conteúdo validado com pelo menos 90% de concordância entre os juízes especialistas. Conclui-se que a validação de conteúdo de um protocolo assistencial permite alcançar credibilidade científica para os cuidados.

Dentre os nove itens propostos por Pasquali^(13)^, destacaram-se neste estudo os itens de atualização e coerência, com IVC de 1,00 quando analisados individualmente em todos os domínios. Um estudo recente apontou que, apesar da utilização de diferentes métodos, a revisão da literatura científica permite agregar as melhores e mais atuais evidências disponíveis para identificar práticas eficientes na saúde^(25)^. Esses apontamentos podem ter influenciado o alto índice de IVC e o alfa de Cronbach alcançados em todos os domínios quando avaliados individualmente.

Outros itens bastante valorizados pelos juízes dizem respeito à abrangência, clareza, criticidade dos itens, objetividade e redação científica. A redação científica de instrumentos assistenciais é uma etapa crucial, considerando que todo instrumento ou protocolo assistencial deve ser redigido de forma a evitar qualquer ambiguidade, incompreensão, vagueza ou subjetividade^(22)^.

A relevância e a sequência também foram validadas com um bom IVC, e o alfa de Cronbach atingiu 0,85. É fundamental que o conteúdo se concentre na temática proposta, com uma sequência lógica de ideias, desde questões mais gerais até assuntos específicos^(15)^.

A utilização do *Google Forms* como ferramenta apresentou diversas vantagens, porém, as principais desvantagens incluem a elaboração do questionário, a abstenção de participantes e o longo tempo de execução. Vários estudos^(30,31)^ utilizaram essa ferramenta para a coleta de dados, corroborando o maior alcance de profissionais nesta pesquisa. Além disso, foi possível padronizar as respostas para que respostas similares fossem consideradas equivalentes no momento da análise^(32)^. Assim, é importante homogeneizar as perguntas, o conjunto de participantes e o ambiente de aplicação do questionário, a fim de evitar influências nas respostas.

### Limitações do estudo

As dificuldades em sensibilizar os juízes da amostra por conveniência foi uma limitação observada. Verificou-se uma taxa de retorno e de resposta reduzida, indo ao encontro do que é apontado nas desvantagens em realizar coleta de dados pela Internet. Ademais, a escassez de estudos de validação de tecnologias como protocolos assistenciais à gestante de alto risco dificultou a comparação e discussão dos resultados.

### Contribuições para a área da Enfermagem e Saúde Pública

A divulgação desse estudo permite o preenchimento de uma lacuna percebida. Logo, espera-se que a presente pesquisa possa contribuir com iniciativas direcionadas a ampliar as possibilidades de uma assistência segura e completa dos profissionais envolvidos no atendimento a gestante de alto risco, a partir do uso de novas tecnologias, a exemplo dos protocolos assistenciais.

## CONCLUSÕES

O protocolo assistencial interprofissional de cuidado pré-natal especializado contemplou temáticas relacionadas ao pré-natal de alto risco no CAEMI, acolhimento das gestantes, fluxograma do circuito de atendimento, atribuições específicas da equipe interprofissional, atribuições coletivas da equipe interprofissional, o fluxo de atendimento da gestante de alto risco na rede de atenção a saúde e os atributos para a prática interprofissional colaborativa.

Constatou-se que o protocolo é válido quanto ao conteúdo, com alto grau de recomendação pelos juízes especialistas para ser utilizado por uma equipe interdisciplinar às gestantes de alto risco. Os dados reforçam que o material validado contribuirá para a prática clínica no que tange sistematizar, organizar e estruturar o cuidado, promovendo melhorias na qualidade da assistência pré-natal de alto risco e norteando equipes interdisciplinares.

Por se tratar de um estudo pioneiro na construção e validação de um protocolo assistencial às gestantes de alto risco, este estudo trará benefícios para o desenvolvimento de novas tecnologias assistenciais validadas e sua ampla divulgação na comunidade científica. Recomenda-se a realização de futuros estudos para a aplicação e avaliação do protocolo.
